# Reporter-Based Screens for the Ubiquitin/Proteasome System

**DOI:** 10.3389/fchem.2020.00064

**Published:** 2020-02-11

**Authors:** Maria E. Gierisch, Tatiana A. Giovannucci, Nico P. Dantuma

**Affiliations:** Department of Cell and Molecular Biology, Karolinska Institutet, Stockholm, Sweden

**Keywords:** reporter assay, ubiquitin-proteasome system, high-content screen, high-throughput drug screening, proteolysis

## Abstract

Instant and adequate handling of misfolded or otherwise aberrant proteins is of paramount importance for maintaining protein homeostasis in cells. The ubiquitin/proteasome system (UPS) is a central player in protein quality control as it operates in a seek-and-destroy mode, thereby facilitating elimination of faulty proteins. While proteasome inhibition is in clinical use for the treatment of hematopoietic malignancies, stimulation of the UPS has been proposed as a potential therapeutic strategy for various neurodegenerative disorders. High-throughput screens using genetic approaches or compound libraries are powerful tools to identify therapeutic intervention points and novel drugs. Unlike assays that measure specific activities of components of the UPS, reporter substrates provide us with a more holistic view of the general functional status of the UPS in cells. As such, reporter substrates can reveal new ways to obstruct or stimulate this critical proteolytic pathway. Here, we discuss various reporter substrates for the UPS and their application in the identification of key players and the pursuit for novel therapeutics.

## Targeting The Ubiquitin/Proteasome System (Ups)

**Figure 1:**
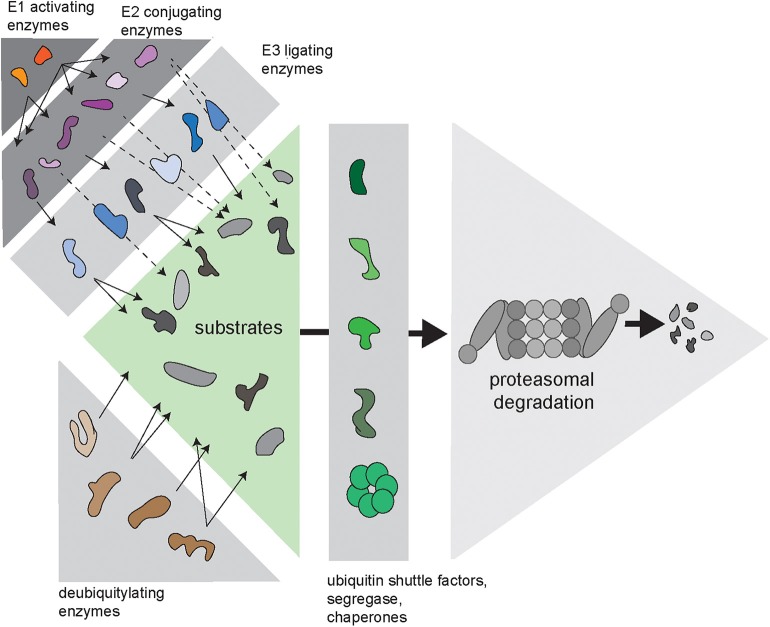
Complexity of the ubiquitin/proteasome system (UPS). Via an enzymatic cascade consisting of E1 ubiquitin-activating enzymes, E2 ubiquitin-conjugating enzymes and E3 ubiquitin ligases, ubiquitin is transferred to substrate proteins. E2-bound ubiquitin can be passed on to an E3, which transfers it to the substrate (continuous arrows), or via E2–E3 complexes, where the E2 transfers the ubiquitin to the E3-bound substrate (dashed arrows). DUBs remove ubiquitin chains from proteins, which can either promote or inhibit degradation of substrates. Substrates are transferred to the proteasome by various ubiquitin shuttle factors and often require handling by the ubiquitin-selective seggregase VCP.

Cells need to balance production, maintenance and degradation of their proteome throughout their entire lifespan, which may vary depending on the type of cell from hours to decades. Proteins that fail protein quality control are a potential risk for protein homeostasis and are targeted for destruction by the ubiquitin/proteasome system (UPS) (Ciechanover, 2005). Proteasome-mediated degradation is initiated by conjugation of the protein modifier ubiquitin to lysine residues of proteins designated for destruction. This initial ubiquitin moiety can be used for the assembly of ubiquitin chains, which are formed through ubiquitylation of one out of seven internal lysine residues in ubiquitin (Komander and Rape, 2012). Conjugation of ubiquitin to substrates is regulated by an enzymatic cascade consisting of E1 ubiquitin-activating enzymes, E2 ubiquitin-conjugating enzymes, and E3 ubiquitin ligases (Figure 1). In some cases, specific E4 ubiquitin chain elongators are involved in extending the ubiquitin chains on substrates (Koegl et al., 1999). Subsequently, proteins are degraded into small peptide fragments inside the proteolytic chamber of proteasomes (Bard et al., 2018). Ubiquitylated substrates often require the unfoldase activity of the ubiquitin-targeted segregase valosin-containing protein (VCP), also known as p97, before they can be processed by the proteasome (Twomey et al., 2019). Moreover, several ubiquitin shuttle factors are responsible for the delivery of the substrate to the proteasome (Elsasser and Finley, 2005). To allow efficient degradation, ubiquitin chains have to be removed prior to degradation, which is facilitated by the POH1 deubiquitylating enzyme (DUB) at the entrance of the proteasome (Verma et al., 2002; Yao and Cohen, 2002). In contrast, the proteasome-associated DUB USP14 can rescue proteins from degradation by removing ubiquitin chains before the proteasome has initiated degradation (Kraut et al., 2007).

Due to their hyperactive state and compromised genome integrity, cancer cells produce elevated levels of aberrant proteins. This phenomenon is believed to make them more susceptible to drugs that restrict the activity of the UPS (Bruning and Juckstock, 2015). Pharmacological inhibition of the UPS is typically accomplished by targeting the proteasome (Kisselev and Goldberg, 2001). Bortezomib was the first FDA-approved proteasome inhibitor and currently serves as a drug for first-line treatment of multiple myeloma and mantle cell lymphoma (Adams, 2004). Despite the fact that the successful introduction of proteasome inhibition for treatment of hematopoietic malignancies has provided the field with an encouraging proof-of-principle, other therapeutic strategies for UPS inhibition remain in an early exploratory stage. The few clinically approved UPS-targeting drugs are rather crude in their action as they all target the main chymotrypsin-like activity of the proteasome (Fricker, 2019). Concerns regarding the observed adverse effects, the development of resistance against proteasome inhibitors and the poor activity of proteasome inhibitors toward solid tumors are strong arguments for the development of drugs directed against other targets within the UPS.

On the other hand, enhancing UPS activity may be desirable in diseases where accumulation of misfolded proteins is responsible for cellular dysfunction and decay, which is the case for a broad variety of neurodegenerative diseases characterized by accumulation of protein aggregates, such as Alzheimer's, Parkinson's and Huntington's disease as well as amyotrophic lateral sclerosis (Boland et al., 2018). As most compounds block catalytic activities of enzymes, pharmacological stimulation might potentially be more challenging. However, due to the complex nature of the UPS, overall stimulation may be feasible through inhibition of specific enzymes that slow down the process. In line with this notion, it has been shown that the USP14 inhibitor IU1 stimulates the degradation of aggregation-prone proteins, such as tau and TDP-43, both linked to neurodegenerative diseases (Lee et al., 2010).

One of the most daunting tasks in the development of new modulators of the UPS is the identification of proteins and processes that can be targeted. More than 40 E2 conjugation enzymes can pair with over 600 different E3 ligases, while around 100 DUBs are involved in the removal of ubiquitin chains. Moreover, a vast number of proteins is involved in coordinating this process, guiding substrates to the proteasome and prepare them for efficient degradation. It is hard to predict how inhibition of individual players will affect the overall efficacy of the UPS. Screening campaigns are often designed to interrogate the activity of a specific enzymatic target, which requires a preselection of a target-of-interest. An alternative approach are phenotypic assays that are based on the ectopic expression of engineered fluorescent UPS substrates, which lack a biological function but can be readily and quantitatively detected by their fluorescence (Neefjes and Dantuma, 2004). The latter assays are unbiased and allow the identification of novel ways to modulate UPS activity without requiring *a priori* knowledge on the mode of action of the targets.

## Ups Reporter Substrates

**Figure 2:**
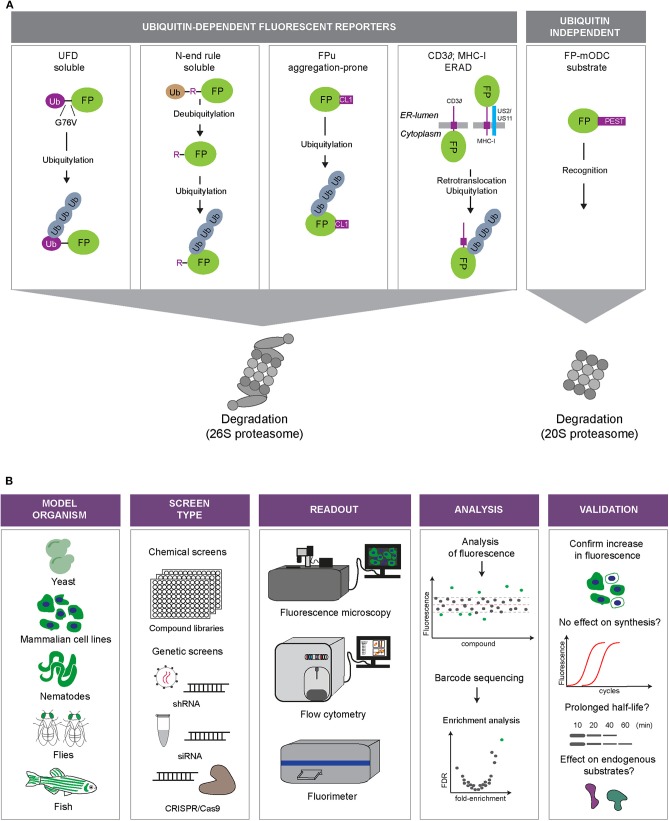
Fluorescent UPS reporters and screening strategies. **(A)** UPS reporters. Fluorescent proteins (FP) are converted into UPS reporter substrates through introduction of degradation signals of different nature (depicted in purple). The different reporter proteins allow monitoring of different UPS activities: degradation of soluble proteins (Ub^G76V^-FP and Ub-R-FP), degradation of aggregation-prone proteins (FP-CL1), ER-associated degradation (ERAD; MHC-I-FP and CD3δ-FP) or ubiquitin-independent degradation (FP-mODC). **(B)** Overview of the steps involved in high-throughput screens using fluorescent reporters of the UPS. Model: Fluorescent reporters are expressed in cell or animal models suited for high-throughput screening. Screening format: Compound libraries or genetic libraries can be used. Modulation of genetic expression can be achieved via siRNA, shRNA, or CRISPR/Cas9 approaches. Readout: Fluorescence microscopy, flow cytometry or fluorimetry can be used as a fluorescence readout or for sorting a specific cell population by fluorescence activated cell sorting (FACS). Analysis: Hits can be identified through readout of fluorescence intensity or sequencing of selected cells. Validation: Examples of various methods that can be used to validate hits.

UPS reporter substrates are based on targeting an otherwise stable protein for proteasomal degradation through the introduction of a degradation signal (Neefjes and Dantuma, 2004) (Figure 2A). Degradation signals, so-called degrons, are conserved motifs that target proteins for proteasomal degradation. One of the first identified degrons is the N-terminal amino acid of proteins (Bachmair et al., 1986). This was discovered by expressing fusion proteins with an N-terminal ubiquitin moiety, which will be proteolytically cleaved in cells, leaving the C-terminal protein with an amino terminus that corresponds to the sequence following the DUB cleavage site. Depending on the nature of the new N-terminal amino acid, it may function as a degron that recruits ubiquitin ligases and determines the half-life of the protein.

When the DUB cleavage of the N-terminal ubiquitin was prevented by substituting the final glycine of ubiquitin to valine (G76V), proteins were still found to be destabilized, but this time another set of proteins was involved in their recognition and degradation (Johnson et al., 1992). In these fusions, the uncleavable N-terminal ubiquitin is marked with ubiquitin chains that target it for proteasomal degradation (Johnson et al., 1995). This type of engineered proteins are known as ubiquitin fusion degradation (UFD) substrates.

Both the N-end rule and UFD degradation signals are versatile motifs that can be used to target most proteins-of-interest for degradation. Both degrons were used for the development of the first green fluorescent protein (GFP)-based reporter substrates that were expressed in cells (Dantuma et al., 2000) and mice (Lindsten et al., 2003). Expression of a luciferase carrying multiple UFD signals enabled also *in vivo* analysis of the effect of drugs on UPS activity in xenograft transplants in mice (Luker et al., 2003), while a UFD-targeted version of a photoconvertable fluorescent protein allowed determination of the half-lives of UPS substrates in living nematodes (Hamer et al., 2010), illustrating the potential of this approach.

Another engineered degradation signal that has been used for the generation of reporter substrates is a short C-terminal linkage (CL) referred to as CL1. The CL1 peptide was identified in a yeast screen aimed at identifying peptide extensions that degrade proteins dependent on endoplasmic reticulum (ER)-anchored ubiquitin-conjugating enzymes involved in marking misfolded ER proteins for proteasomal degradation (Gilon et al., 1998, 2000). C-terminal tagging of GFP with CL1 resulted in a short-lived GFP, which has been used for generating cellular (Bence et al., 2001) and mouse UPS models (Bove et al., 2006; Liu et al., 2006). Fluorescent proteins destabilized by CL1 tend to aggregate most probably due to its hydrophobic nature (Menéndez-Benito et al., 2005; Link et al., 2006). As such, GFP-CL1 and related fluorescent reporters may be in particular suited to probe into the ability of cells to eliminate aggregation-prone proteins by proteasomal degradation.

In addition to these engineered motifs, naturally occurring degradation signals have also been exploited to destabilize reporter proteins. Fluorescent proteins have been provided with natural degradation signals or fused to full-length proteasome substrates. A natural motif used for this purpose is the PEST sequence of ornithine decarboxylase (ODC). The ODC degradation signal has the special feature that it can target proteins for ubiquitin-independent proteasomal degradation, thereby bypassing the complex machinery for ubiquitylation (Hoyt et al., 2005).

Other full-length proteins that have been used for the generation of reporter substrates are the heavy chain (HC) of major histocompatibility complex (MHC) class I molecules, which, in the presence of the viral proteins US2 or US11, is rapidly dislocated from the ER into the cytosol, ubiquitylated and disposed by the proteasome (Schust et al., 1998). MHC-HC shares this pathway with other proteins targeted for ER-associated degradation (ERAD) (Berner et al., 2018). Another commonly used ERAD reporter substrate is the T cell receptor subunit CD3δ. When expressed in other cells than T lymphocytes, CD3δ fusions are orphan subunits unable to find their binding partner, resulting in targeting of these fusions to the ERAD pathway (Yang et al., 1998).

While MHC class I and CD3δ require ERAD proteins that facilitate identification of the reporter as aberrant in the ER and its translocation into the cytosol, the CL1-destabilized reporter substrates, which are cytosolic reporters, will only engage the final steps of the ERAD pathway. Thus, different UPS reporters display differential sensitivities for different branches of the UPS: the N-end rule and UFD substrates behave as soluble, properly folded proteins, CL1 mimics aggregation-prone proteins, CD3δ and MHC class I HC are ERAD substrates and ODC-destabilized proteins report on the status of ubiquitin-independent degradation.

## Ups Reporter-Based Screens

A number of genetic and compound screens have been published in which the usage of UPS reporters played a central role. In these screens, transiently transfected or stably integrated reporters in mammalian cells served as read-outs for global changes in the UPS in high-throughput screens for genetic or chemical modulators (Figure 2B).

Phenotypic assays based on reporter substrates have been used for the discovery of novel inhibitory compounds. A ChemBridge library consisting of around 16,000 compounds was screened using a cell line expressing the HC of enhanced green fluorescent protein (EGFP)-tagged MHC-I HC (EGFP-HC) and viral US2, resulting in targeting EGFP-HC for ERAD. This screen resulted in the identification of two structurally-related inhibitory compounds: Eeyarestatin 1 (Eer1) and 2 (Eer2) (Fiebiger et al., 2004). Follow-up studies revealed that Eer1 blocks EGFP-HC degradation by interfering with VCP-mediated segregation and suggested that this may be due to inhibition of the VCP-associated DUB ataxin-3 (Wang et al., 2008). In line with the model that Eer1 interferes with the function of VCP, Eer1 induced accumulation of a VCP-dependent reporter, while it did not interfere in the degradation of a VCP-independent substrate (Chou and Deshaies, 2011).

In another screening campaign for UPS inhibitors, the library of pharmacological active compounds (LOPAC) was tested in a high-throughput format using cells that expressed the ubiquitin-independent substrate ZsGreen-ODC. Surprisingly, disulfiram, an FDA-approved drug for treatment of alcohol addiction, was found to inhibit UPS activity and displayed cytotoxic effects on a myeloma cell line (Rickardson et al., 2007). Disulfiram had been previously found to interfere with NF-κB activity (Wang et al., 2003). This was later pinpointed to copper-dependent inhibition of the proteasome, whose activity is required for NF-κB translocation (Chen et al., 2006). In a more recent study, the ditiocarb-copper complex, a metabolite of disulfiram, was shown to also impair degradation of a UFD reporter substrate via inhibition of the VCP-adaptor protein NPL4 (Skrott et al., 2017), suggesting that disulfiram may modulate several targets within the UPS.

Genetic UPS screens are commonly based on manipulation of the gene expression in reporter cells using siRNA, shRNA or CRISPR/Cas9 technology. Cells expressing a UFD-destabilized reporter were used in a screen aimed at identifying proteins involved in the mammalian UFD pathway (Poulsen et al., 2012). An siRNA-based library targeting 558 genes was used in this screen. This led to the identification of several UFD components including HUWE1, a HECT domain ubiquitin ligase. A natural substrate of HUWE1 is UBB^+1^, an aberrant ubiquitin found in neurological and non-neurological protein misfolding disorders (Van Leeuwen et al., 1998). UBB^+1^ has an uncleavable N-terminal ubiquitin moiety and resembles artificial UFD substrates (Lindsten et al., 2002). It is noteworthy that HUWE is overexpressed in lung, breast and colon carcinoma, suggesting also a possible role in tumorigenesis (Adhikary et al., 2005; Yoon et al., 2005; Kao et al., 2018).

A genome-wide CRISPR/Cas9 library was screened using a GFP-CL1-expressing cell line. Fluorescence-activated cell sorting (FACS) was employed to obtain a population enriched for cells with elevated GFP-CL1 levels, which were subsequently analyzed by barcode sequencing (Leto et al., 2019). This resulted in the identification of new genes of the ERAD ubiquitin conjugation machinery, including the ubiquitin ligase RNF139/TRC8 and ubiquitin-conjugating enzyme UBE3C. In a different screen, the near-haploid cell line KBM7 (Carette et al., 2009), stably expressing mCherry-CL1 was used to sort mCherry^High^ cells by flow cytometry after insertional mutagenesis with a gene-trapping retrovirus (Stefanovic-Barrett et al., 2018). In addition to RNF139/TRC8, a second ER-resident E3 ligases, MARCH6, was found to function in ubiquitin-dependent degradation of soluble and tail anchored ER proteins.

In a UPS-specific and genome-wide siRNA-based screen, a fluorescently tagged, thermally unstable nuclear reporter was used to identify proteins involved in nuclear protein quality control (Pegoraro et al., 2012). The screen was performed by analyzing 384-well plates with automated fluorescence microscopy. Besides a number of hits that were anticipated, such as proteasome subunits, they found the proteasome assembly chaperone POMP (Burri et al., 2000; Fricke et al., 2007), and eIF3, a translation initiation complex (Abbott and Proud, 2004), to be important for efficient nuclear protein quality control.

Upon construction of two novel shRNA-based libraries, the fluorescent reporter ZsGreen-mODC was used to validate the functionality of these libraries (Paddison et al., 2004; Silva et al., 2005). Cells were transfected with an expression vector for the ZsGreen-mODC reporter together with either a plasmid library consisting of 7,000 unique shRNAs (Paddison et al., 2004) or a sub-library consisting of shRNAs specific for a large number of kinases and proteasome subunits shRNAs (Silva et al., 2005). The performance of the library and setup of the screen was confirmed as shRNAs directed against proteasome subunits were readily identified by accumulation of the reporter substrate. In this particular case, the UPS reporter were used as a fast and robust tool for validation of the screening libraries.

## Concluding Remarks

With the appearance of advanced techniques and equipment, high-throughput and high-content screenings have become attractive approaches for addressing biological questions and drug development. Many different fluorescent substrate reporters have emerged over the years in parallel with a better understanding of the UPS and an increased awareness of the UPS as therapeutic target. A number of opportunities for optimizing these reporter assays and tailoring them to specific purposes remain. Although some UPS reporter mouse strains have been generated, it is obvious that mouse models are not suited for large-scale screening efforts. However, other animal reporter models, such as nematodes (Hamer et al., 2010), fruit flies (Pandey et al., 2007), and zebrafish (Imamura et al., 2012), open possibilities to perform genetic and compound screens on a larger scale in *in vivo* models.

In addition to UPS inhibitors, there is an emerging interest for UPS stimulators, which may be harder to identify with the currently available assays. Hence, there is a need for the development of novel reporters that are more suited for detecting an increase in UPS activity. A point of improvement may be the use of internal stable reference proteins, which have been already applied in some screens (Yen and Elledge, 2008; Yen et al., 2008; Wu et al., 2016). These reference proteins can correct for differences in synthesis of the reporter and may give a more robust readout, thereby reducing the number of false hits. Due to the relatively low steady-state levels of the presently available reporter substrates, detection of enhanced degradation in high content screens may be problematic, even in the presence of a stable reference protein. Two recent studies elegantly overcame this limitation by creating a system in which the expression of the reporter protein is repressed by a transcriptional regulator (Zhao et al., 2014; Zeng et al., 2019). Fusion of the transcriptional repressor to a destabilizing signal results in an inverse correlation between the activity of the UPS and the levels of the reporter protein that are regulated by the repressor. However, the applicability of these reporter systems in high-throughput campaigns remains to be validated.

Genetic screens and compound screens each have their own strengths and weaknesses and can complement each other in screening campaigns. Genetic screens may result in the identification of interesting but poorly druggable candidates, while compound screens may identify powerful drug-like compounds but encounter difficulties in target identification. Upon combination of these complementary approaches and adapting the assays to more disease-relevant settings, fluorescent reporters can be used to their full potential in the pursuit for novel ways of modulating the UPS in human diseases.

## Author Contributions

MG, TG, and ND wrote the manuscript.

### Conflict of Interest

The authors declare that the research was conducted in the absence of any commercial or financial relationships that could be construed as a potential conflict of interest.

## References

[B1] Abbott C. M. Proud C. G. (2004). Translation factors: in sickness and in health. Trends Biochem. Sci. 29, 25–31. 10.1016/j.tibs.2003.11.006 14729329

[B2] Adams J. (2004). The development of proteasome inhibitors as anticancer drugs. Cancer Cell 5, 417–421. 10.1016/S1535-6108(04)00120-5 15144949

[B3] Adhikary S. Marinoni F. Hock A. Hulleman E. Popov N. Beier R.. (2005). The ubiquitin ligase HectH9 regulates transcriptional activation by Myc and is essential for tumor cell proliferation. Cell 123, 409–421. 10.1016/j.cell.2005.08.016 16269333

[B4] Bachmair A. Finley D. Varshavsky A. (1986). *In vivo* half-life of a protein is a function of its amino-terminal residue. Science 234, 179–186. 10.1126/science.3018930 3018930

[B5] Bard J. A. M. Goodall E. A. Greene E. R. Jonsson E. Dong K. C.. (2018). Structure and Function of the 26S Proteasome. Annu. Rev. Biochem. 87, 697–724. 10.1146/annurev-biochem-062917-011931 29652515 PMC6422034

[B6] Bence N. F. Sampat R. M. Kopito R. R. (2001). Impairment of the ubiquitin-proteasome system by protein aggregation. Science 292, 1552–1555. 10.1126/science.292.5521.1552 11375494

[B7] Berner N. Reutter K. R. Wolf D. H. (2018). Protein quality control of the endoplasmic reticulum and ubiquitin-proteasome-triggered degradation of aberrant proteins: yeast pioneers the path. Annu. Rev. Biochem. 87, 751–782. 10.1146/annurev-biochem-062917-012749 29394096

[B8] Boland B. Yu W. H. Corti O. Mollereau B. Henriques A. Bezard E.. (2018). Promoting the clearance of neurotoxic proteins in neurodegenerative disorders of ageing. Nat. Rev. Drug. Discov. 17, 660–688. 10.1038/nrd.2018.109 30116051 PMC6456907

[B9] Bove J. Zhou C. Jackson-Lewis V. Taylor J. Chu Y. Rideout H. J.. (2006). Proteasome inhibition and Parkinson's disease modeling. Ann. Neurol. 60, 260–264. 10.1002/ana.20937 16862585

[B10] Bruning A. Juckstock J. (2015). Misfolded proteins: from little villains to little helpers in the fight against cancer. Front. Oncol. 5:47. 10.3389/fonc.2015.00047 25759792 PMC4338749

[B11] Burri L. Hockendorff J. Boehm U. Klamp T. Dohmen R. J. Levy F. (2000). Identification and characterization of a mammalian protein interacting with 20S proteasome precursors. Proc. Natl. Acad. Sci. U.S.A. 97, 10348–10353. 10.1073/pnas.190268597 10973495 PMC27027

[B12] Carette J. E. Guimaraes C. P. Varadarajan M. Park A. S. Wuethrich I. Godarova A.. (2009). Haploid genetic screens in human cells identify host factors used by pathogens. Science 326, 1231–1235. 10.1126/science.1178955 19965467

[B13] Chen D. Cui Q. C. Yang H. Dou Q. P. (2006). Disulfiram, a clinically used anti-alcoholism drug and copper-binding agent, induces apoptotic cell death in breast cancer cultures and xenografts via inhibition of the proteasome activity. Cancer Res. 66, 10425–10433. 10.1158/0008-5472.CAN-06-2126 17079463

[B14] Chou T.-F. Deshaies R. J. (2011). Quantitative cell-based protein degradation assays to identify and classify drugs that target the ubiquitin-proteasome system. J. Biol. Chem. 286, 16546–16554. 10.1074/jbc.M110.215319 21343295 PMC3089497

[B15] Ciechanover A. (2005). Proteolysis: from the lysosome to ubiquitin and the proteasome. Nat. Rev. Mol. Cell Biol. 6:79. 10.1038/nrm1552 15688069

[B16] Dantuma N. P. Lindsten K. Glas R. Jellne M. Masucci M. G. (2000). Short-lived green fluorescent proteins for quantifying ubiquitin/proteasome-dependent proteolysis in living cells. Nat. Biotechnol. 18, 538–543. 10.1038/75406 10802622

[B17] Elsasser S. Finley D. (2005). Delivery of ubiquitinated substrates to protein-unfolding machines. Nat. Cell Biol. 7, 742–749. 10.1038/ncb0805-742 16056265

[B18] Fiebiger E. Hirsch C. Vyas J. M. Gordon E. Ploegh H. L. Tortorella D. (2004). Dissection of the dislocation pathway for type I membrane proteins with a new small molecule inhibitor, eeyarestatin. Mol. Biol. Cell 15, 1635–1646. 10.1091/mbc.e03-07-0506 14767067 PMC379262

[B19] Fricke B. Heink S. Steffen J. Kloetzel P. M. Kruger E. (2007). The proteasome maturation protein POMP facilitates major steps of 20S proteasome formation at the endoplasmic reticulum. EMBO Rep. 8, 1170–1175. 10.1038/sj.embor.7401091 17948026 PMC2267243

[B20] Fricker L. D. (2019). Proteasome inhibitor drugs. Annu. Rev. Pharmacol. Toxicol. 10.1146/annurev-pharmtox-010919-023603 31479618

[B21] Gilon T. Chomsky O. Kulka R. G. (1998). Degradation signals for ubiquitin system proteolysis in Saccharomyces cerevisiae. EMBO J. 17, 2759–2766. 10.1093/emboj/17.10.2759 9582269 PMC1170616

[B22] Gilon T. Chomsky O. Kulka R. G. (2000). Degradation signals recognized by the Ubc6p-Ubc7p ubiquitin-conjugating enzyme pair. Mol. Cell Biol. 20, 7214–7219. 10.1128/MCB.20.19.7214-7219.2000 10982838 PMC86275

[B23] Hamer G. Matilainen O. Holmberg C. I. (2010). A photoconvertible reporter of the ubiquitin-proteasome system *in vivo*. Nat. Methods 7:473. 10.1038/nmeth.1460 20453865

[B24] Hoyt M. A. Zhang M. Coffino P. (2005). Probing the ubiquitin/proteasome system with ornithine decarboxylase, a ubiquitin-independent substrate. Methods Enzymol. 398, 399–413. 10.1016/S0076-6879(05)98033-6 16275346

[B25] Imamura S. Yabu T. Yamashita M. (2012). Protective role of cell division cycle 48 (CDC48) protein against neurodegeneration via ubiquitin-proteasome system dysfunction during zebrafish development. J. Biol. Chem. 287, 23047–23056. 10.1074/jbc.M111.332882 22549779 PMC3391108

[B26] Johnson E. S. Bartel B. Seufert W. Varshavsky A. (1992). Ubiquitin as a degradation signal. EMBO J. 11, 497–505. 10.1002/j.1460-2075.1992.tb05080.x 1311250 PMC556480

[B27] Johnson E. S. Ma P. C. Ota I. M. Varshavsky A. (1995). A proteolytic pathway that recognizes ubiquitin as a degradation signal. J. Biol. Chem. 270, 17442–17456. 10.1074/jbc.270.29.17442 7615550

[B28] Kao S. H. Wu H. T. Wu K. J. (2018). Ubiquitination by HUWE1 in tumorigenesis and beyond. J. Biomed. Sci. 25:67. 10.1186/s12929-018-0470-0 30176860 PMC6122628

[B29] Kisselev A. F. Goldberg A. L. (2001). Proteasome inhibitors: from research tools to drug candidates. Chem. Biol. 8, 739–758. 10.1016/S1074-5521(01)00056-4 11514224

[B30] Koegl M. Hoppe T. Schlenker S. Ulrich H. D. Mayer T. U. Jentsch S. (1999). A novel ubiquitination factor, E4, is involved in multiubiquitin chain assembly. Cell 96, 635–644. 10.1016/S0092-8674(00)80574-7 10089879

[B31] Komander D. Rape M. (2012). The ubiquitin code. Annu. Rev. Biochem. 81, 203–229. 10.1146/annurev-biochem-060310-170328 22524316

[B32] Kraut D. A. Prakash S. Matouschek A. (2007). To degrade or release: ubiquitin-chain remodeling. Trends Cell Biol. 17, 419–421. 10.1016/j.tcb.2007.06.008 17900906

[B33] Lee B. H. Lee M. J. Park S. Oh D. C. Elsasser S. Chen P. C.. (2010). Enhancement of proteasome activity by a small-molecule inhibitor of USP14. Nature 467, 179–184. 10.1038/nature09299 20829789 PMC2939003

[B34] Leto D. E. Morgens D. W. Zhang L. Walczak C. P. Elias J. E. Bassik M. C.. (2019). Genome-wide CRISPR analysis identifies substrate-specific conjugation modules in ER-associated degradation. Mol. Cell 73, 377–389 e311. 10.1016/j.molcel.2018.11.015 30581143 PMC6338494

[B35] Lindsten K. De Vrij F. M. Verhoef L. G. Fischer D. F. Van Leeuwen F. W. Hol E. M.. (2002). Mutant ubiquitin found in neurodegenerative disorders is a ubiquitin fusion degradation substrate that blocks proteasomal degradation. J. Cell. Biol. 157, 417–427. 10.1083/jcb.200111034 11980917 PMC2173284

[B36] Lindsten K. Menéndez-Benito V. Masucci M. G. Dantuma N. P. (2003). A transgenic mouse model of the ubiquitin/proteasome system. Nat. Biotechnol. 21:897. 10.1038/nbt851 12872133

[B37] Link C. D. Fonte V. Hiester B. Yerg J. Ferguson J. Csontos S.. (2006). Conversion of green fluorescent protein into a toxic, aggregation-prone protein by C-terminal addition of a short peptide. J. Biol. Chem. 281, 1808–1816. 10.1074/jbc.M505581200 16239215

[B38] Liu J. Chen Q. Huang W. Horak K. M. Zheng H. Mestril R.. (2006). Impairment of the ubiquitin-proteasome system in desminopathy mouse hearts. FASEB J. 20, 362–364. 10.1096/fj.05-4869fje 16371426

[B39] Luker G. D. Pica C. M. Song J. Luker K. E. Piwnica-Worms D. (2003). Imaging 26S proteasome activity and inhibition in living mice. Nat. Med. 9, 969–973. 10.1038/nm894 12819780

[B40] Menéndez-Benito V. Verhoef L. G. G. C. Masucci M. G. Dantuma N. P. (2005). Endoplasmic reticulum stress compromises the ubiquitin–proteasome system. Hum. Mol. Genet. 14, 2787–2799. 10.1093/hmg/ddi312 16103128

[B41] Neefjes J. Dantuma N. P. (2004). Fluorescent probes for proteolysis: tools for drug discovery. Nat. Rev. Drug Discov. 3, 58–69. 10.1038/nrd1282 14708021 PMC7097193

[B42] Paddison P. J. Silva J. M. Conklin D. S. Schlabach M. Li M. Aruleba S.. (2004). A resource for large-scale RNA-interference-based screens in mammals. Nature 428, 427–431. 10.1038/nature02370 15042091

[B43] Pandey U. B. Nie Z. Batlevi Y. Mccray B. A. Ritson G. P. Nedelsky N. B.. (2007). HDAC6 rescues neurodegeneration and provides an essential link between autophagy and the UPS. Nature 447, 859–863. 10.1038/nature05853 17568747

[B44] Pegoraro G. Voss T. C. Martin S. E. Tuzmen P. Guha R. Misteli T. (2012). Identification of mammalian protein quality control factors by high-throughput cellular imaging. PLoS ONE 7:e31684. 10.1371/journal.pone.0031684 22363705 PMC3282772

[B45] Poulsen E. G. Steinhauer C. Lees M. Lauridsen A. M. Ellgaard L. Hartmann-Petersen R. (2012). HUWE1 and TRIP12 collaborate in degradation of ubiquitin-fusion proteins and misframed ubiquitin. PLoS ONE 7:e50548. 10.1371/journal.pone.0050548 23209776 PMC3507821

[B46] Rickardson L. Wickström M. Larsson R. Lövborg H. (2007). Image-based screening for the identification of novel proteasome inhibitors. J. Biomol. Screen. 12, 203–210. 10.1177/1087057106297115 17208922

[B47] Schust D. J. Tortorella D. Seebach J. Phan C. Ploegh H. L. (1998). Trophoblast class I major histocompatibility complex (MHC) products are resistant to rapid degradation imposed by the human cytomegalovirus (HCMV) gene products US2 and US11. J. Exp. Med. 188, 497–503. 10.1084/jem.188.3.497 9687527 PMC2212475

[B48] Silva J. M. Li M. Z. Chang K. Ge W. Golding M. C. Rickles R. J.. (2005). Second-generation shRNA libraries covering the mouse and human genomes. Nat. Genet. 37, 1281–1288. 10.1038/ng1650 16200065

[B49] Skrott Z. Mistrik M. Andersen K. K. Friis S. Majera D. Gursky J.. (2017). Alcohol-abuse drug disulfiram targets cancer via p97 segregase adaptor NPL4. Nature 552:194. 10.1038/nature25016 29211715 PMC5730499

[B50] Stefanovic-Barrett S. Dickson A. S. Burr S. P. Williamson J. C. Lobb I. T. Van Den Boomen D. J.. (2018). MARCH6 and TRC8 facilitate the quality control of cytosolic and tail-anchored proteins. EMBO Rep. 19:e45603. 10.15252/embr.201745603 29519897 PMC5934766

[B51] Twomey E. C. Ji Z. Wales T. E. Bodnar N. O. Ficarro S. B. Marto J. A.. (2019). Substrate processing by the Cdc48 ATPase complex is initiated by ubiquitin unfolding. Science 365:eaax1033. 10.1126/science.aax1033 31249135 PMC6980381

[B52] Van Leeuwen F. W. De Kleijn D. P. Van Den Hurk H. H. Neubauer A. Sonnemans M. A. Sluijs J. A.. (1998). Frameshift mutants of beta amyloid precursor protein and ubiquitin-B in Alzheimer's and Down patients. Science 279, 242–247. 10.1126/science.279.5348.242 9422699

[B53] Verma R. Aravind L. Oania R. Mcdonald W. H. Yates J. R. III. Koonin E. V.. (2002). Role of Rpn11 metalloprotease in deubiquitination and degradation by the 26S proteasome. Science 298, 611–615. 10.1126/science.1075898 12183636

[B54] Wang Q. Li L. Ye Y. (2008). Inhibition of p97-dependent protein degradation by Eeyarestatin I. J. Biol. Chem. 283, 7445–7454. 10.1074/jbc.M708347200 18199748 PMC2276333

[B55] Wang W. Mcleod H. L. Cassidy J. (2003). Disulfiram-mediated inhibition of NF-κB activity enhances cytotoxicity of 5-fluorouracil in human colorectal cancer cell lines. Int. J. Cancer 104, 504–511. 10.1002/ijc.10972 12584750

[B56] Wu Y. Zhou L. Wang X. Lu J. Zhang R. Liang X.. (2016). A genome-scale CRISPR-Cas9 screening method for protein stability reveals novel regulators of Cdc25A. Cell Discov. 2:16014. 10.1038/celldisc.2016.14 27462461 PMC4877570

[B57] Yang M. Omura S. Bonifacino J. S. Weissman A. M. (1998). Novel aspects of degradation of T cell receptor subunits from the endoplasmic reticulum (ER) in T cells: importance of oligosaccharide processing, ubiquitination, and proteasome-dependent removal from ER membranes. J. Exp. Med. 187, 835–846. 10.1084/jem.187.6.835 9500786 PMC2212191

[B58] Yao T. Cohen R. E. (2002). A cryptic protease couples deubiquitination and degradation by the proteasome. Nature 419, 403–407. 10.1038/nature01071 12353037

[B59] Yen H. C. Elledge S. J. (2008). Identification of SCF ubiquitin ligase substrates by global protein stability profiling. Science 322, 923–929. 10.1126/science.1160462 18988848

[B60] Yen H. C. Xu Q. Chou D. M. Zhao Z. Elledge S. J. (2008). Global protein stability profiling in mammalian cells. Science 322, 918–923. 10.1126/science.1160489 18988847

[B61] Yoon S. Y. Lee Y. Kim J. H. Chung A. S. Joo J. H. Kim C. N.. (2005). Over-expression of human UREB1 in colorectal cancer: HECT domain of human UREB1 inhibits the activity of tumor suppressor p53 protein. Biochem. Biophys. Res. Commun. 326, 7–17. 10.1016/j.bbrc.2004.11.004 15567145

[B62] Zeng Y. Bachhav B. Zhao W. Nguyen T. Segatori L. (2019). A hysteretic genetic circuit for detection of proteasomal degradation in mammalian cells. ACS Synth. Biol. 8:2025–35. 10.1021/acssynbio.9b00074 31415719

[B63] Zhao W. Bonem M. Mcwhite C. Silberg J. J. Segatori L. (2014). Sensitive detection of proteasomal activation using the Deg-On mammalian synthetic gene circuit. Nat. Commun. 5:3612. 10.1038/ncomms4612 24710080

